# Oxidative Stress in Fungi: Its Function in Signal Transduction, Interaction with Plant Hosts, and Lignocellulose Degradation

**DOI:** 10.3390/biom5020318

**Published:** 2015-04-03

**Authors:** Michael Breitenbach, Manuela Weber, Mark Rinnerthaler, Thomas Karl, Lore Breitenbach-Koller

**Affiliations:** Department of Cell Biology, Division of Genetics, University of Salzburg, Salzburg 5020, Austria; E-Mails: manuela.weber@stud.sbg.ac.at (M.W.); mark.rinnerthaler@sbg.ac.at (M.R.); Thomas.Karl@sbg.ac.at (T.K.); Hannelore.BREITENBACH-KOLLER@sbg.ac.at (L.B.-K.)

**Keywords:** oxidative stress, NADPH oxidase, peroxiredoxin, protein disulfide isomerase, superoxide radical anion, hydrogen peroxide, hydroxyl radical, mitochondria, integral membrane reductases, lignin degradation

## Abstract

In this review article, we want to present an overview of oxidative stress in fungal cells in relation to signal transduction, interaction of fungi with plant hosts, and lignocellulose degradation. We will discuss external oxidative stress which may occur through the interaction with other microorganisms or plant hosts as well as internally generated oxidative stress, which can for instance originate from NADPH oxidases or “leaky” mitochondria and may be modulated by the peroxiredoxin system or by protein disulfide isomerases thus contributing to redox signaling. Analyzing redox signaling in fungi with the tools of molecular genetics is presently only in its beginning. However, it is already clear that redox signaling in fungal cells often is linked to cell differentiation (like the formation of perithecia), virulence (in plant pathogens), hyphal growth and the successful passage through the stationary phase.

## 1. Introduction: Definition of ROS and Oxidative Stress

Reactive oxygen and nitrogen species (ROS and RNS; often called RONS by a joint generic name) occur in living cells as a consequence of the metabolism of atmospheric oxygen. Most of these molecules are comparatively short-lived and highly reactive, comprising radical as well as non-radical molecular species including singlet oxygen, the superoxide radical anion, hydrogen peroxide, the hydroxyl radical, nitric oxide, peroxynitrite, and other noxious chemical agents derived from the ones just mentioned [1]. A useful overview of the chemistry and biology of those molecules is given by Winterbourn [2]. They cause detrimental chemical changes in proteins, lipids, polysaccharides, DNA, RNA, and even in small metabolites. However, some RONS through the adaptive processes taking place in millions of years of biological evolution, are now being used and active as signaling substances and for metabolic reactions based on radical chemistry which are needed for life and are not *per se* detrimental. ROS are also key players in the interaction of fungi with plant hosts and in the degradation of dead plant materials in the soil.

Living cells are chemically and osmotically isolated from their surroundings creating a electrochemical potential gradient across their plasma membrane, which is necessary for life. The distribution of oxidizing and reducing metabolites in the cell and in the medium creates an inside redox potential of −310 mV relative to the hydrogen electrode under physiological conditions in nearly all living cells [1]. This redox potential is homeostatically controlled by an elaborate system of checks and balances. Deviations from the normal value are tolerated only for a very short time. If they are maintained for some longer time we speak of “oxidative stress” (positive deviation from the normal mean value) or even “reductive stress” (negative deviation from the normal mean value). Both deviations can cause cell death by apoptosis and other processes of programmed cell death like necrosis, which in yeasts and fungi have been studied during the last 15 years, starting with the seminal papers of Madeo *et al.* 1997 [1,3]. Although not absolutely clarified, there is growing evidence that also necrosis is programmed. Therefore the expression of “programmed necrosis” was coined [4]. Strictly speaking, oxidative stress in a cell or cellular compartment is defined by the concentrations of the reduced and oxidized forms of all redox-active metabolites by applying the Nernst equation. In reality, it is often difficult to determine all relevant oxidants and reductants, some of them do not readily participate in redox reactions due to kinetic reasons, and redox exchange between different subcellular compartments further complicates this picture. Another, less rigorous but practically applicable definition of oxidative stress is given by Lushchak [5]: “The situation when due to some reasons the steady-state ROS concentration is acutely or chronically increased leading to oxidative modification of cellular constituents resulting in disturbance of cellular metabolism and regulatory pathways, particularly ROS-based has been called oxidative stress”.

**Figure 1:**
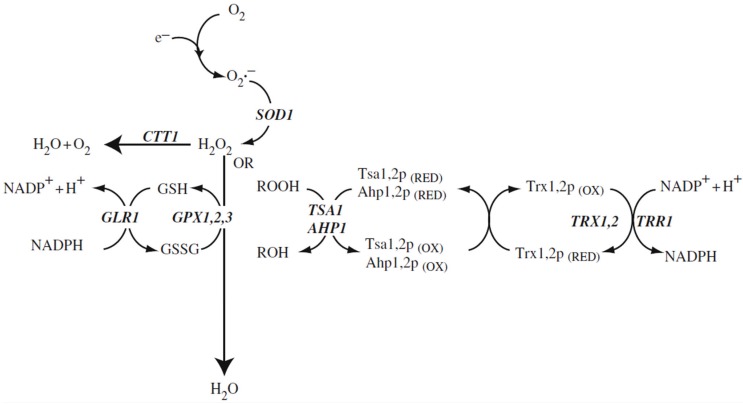
Reactive oxygen species and antioxidant defence systems in the cytosol. The main reactive oxygen species include the superoxide anion radical, hydrogen peroxide, and organic peroxides (ROOH) that are detoxified to water via the Cu, Zn-superoxide dismutase, catalase or glutathione systems. Hydrogen peroxide and organic peroxides (ROOH) can also be detoxified to an alcohol (ROH) by the thioredoxin system. Gene designations are the ones of *S. cerevisiae*. SOD1: Cu, Zn-superoxide dismutase; CTT1: catalase T; GSH: reduced form of glutathione; GSSG: disulfide form of glutathione; GPX1,2,3: the three glutathione peroxidases of *S. cerevisiae*; GLR1: glutathione redutase; TSA1, AHP1: peroxiredoxins; TRX1,2: thioredoxins; TRR1: thioredoxin reductase (after Aung-Htut *et al.* 2012 [1]; with modifications).

The main “redox-buffer”of the cell is the glutathione system, which mediated by a large number of interlinked enzymatic redox systems, can remove ROS and some of their important reaction products like organic hydroperoxides (Figure 1). The number of redox enzymes taking part in these processes even in yeast is over 100 [1] and perhaps 5 times higher in mammalian cells. One key intermediate in these processes is peroxiredoxin, a universal redox protein which we want to describe in more detail below. The necessary reduction equivalents for redox homeostasis are in all cases ultimately supplied by NADPH, which in turn is mainly produced by reducing NADP through the pentose phosphate cycle, one of the oldest metabolic pathways in living cells on earth [6].

The basic biochemistry of oxidative stress and defense against it in fungal cells has been expertly described in recent years [1,5,7,8,9,10,11]. These review aticles include discussions of adaptation to oxidative stress at the level of transcription, postsynthetic modification of proteins, and metabolic reconfiguration and we want to refer the reader to these articles. The basic biochemistry of oxidative stress defense and adaptation is in many respects similar in yeast and in mammalian cells. The main pathways of oxidative stress defense are pictured in Figure 1. We will concentrate here on three topics which in the last few years are increasingly discussed in relation to oxidative stress in fungi: ROS as signal transduction molecules, the role of ROS in the interaction of fungi with plant hosts, and in the degradation of lignocellulose.

Signaling by ROS is a presently highly active field of investigation in mammals, plants and eukaryotic microorganisms [12,13,14]. It becomes more and more clear that the proximate signal-transducing molecule is H_2_O_2_ [12,13,14,15,16] which for the signaling purpose is mostly produced by NADPH oxidases in conjunction with superoxide dismutases (SODs) [16]. We will in the following text give an overview of the oxidative stress created by NADPH oxidases and other metabolic reactions in fungal cells, the role of peroxiredoxins in redox signaling, and the ocurrence and functions of fungal NADPH oxidases.

## 2. Peroxiredoxins

We have decided to review the structure, function , and physiological significance of this class of redox-active enzymes, because they are among the most important oxidative stress defense systems in all cells and have recently been shown to also take part in redox signaling through H_2_O_2_ in eukaryotes. The peroxiredoxins constitute a protein superfamily which has been rather highly conserved throughout evolution [17,18]. In yeast cells as well as in other fungal cells, peroxiredoxins are the quantitatively most abundant redox defense proteins and in some cases make up about 1% of the soluble proteins of the cell [19]. Figure 1 shows the involvement of the yeast peroxiredoxins in the predominant ROS detoxification pathway in the cytoplasm of the cell. Two other pathways are active in addition to the peroxidredoxin pathway: The cytoplasmic catalase (encoded by the gene, *CTT1*) which is specific for H_2_O_2_ and dismutates H_2_O_2_ to H_2_O and O_2_, and the glutathione peroxidase, which is also specific for H_2_O_2_, but reduces it directly to H_2_O mediated by the glutathione (GSH) cycle. The oxidized form of glutathione (GSSG) is ultimately re-reduced using NADPH. We are mentioning in parenthesis that NADPH is the predominant supplier of reduction equivalents in all living cells [6]. The third pathway, based on peroxiredoxins (Figure 1) can reduce a broad spectrum of ROS including H_2_O_2_, alkyl hydroperoxides, lipid hydroperoxides, NO, peroxynitrite, and “unwanted” disulfide bridges in oxidized proteins. The immediate redox partner of peroxiredoxins is thioredoxin, which via thioredoxin reductase is re-reduced, depending again on NADPH as a supply of redution equivalents [1]. This pathway is not only the most abundant at the protein level, it also shows a very high intrinsic enzymatic activity of about 10^7^ M^−1^ sec^−1^ [19]. This is necessary for efficient detoxification given the high toxicity of the peroxides which are scavenged.

Nevertheless, H_2_O_2_ is a preferred signaling substance in eukaryotic cells, as will be discussed below. Toxic peroxides occur in multiple cellular compartments. For efficient detoxification, all these compartments must contain the peroxiredoxin system, as evidenced by the peroxiredoxin isoforms encoded in the yeast and human genome.

In yeast, five isoforms encoded by independent genes are found [1,20]: three of them are found in the cytoplasm (*AHP1*, *TSA2*, *TSA1*) of which *TSA1* is the most important one as evidenced by the strong oxidant hypersensitivity of the corresponding deletion mutant, which is not complemented by the presence of the other isoforms [21,22,23] unless Tsa2 is expressed from an artificial genetic construct using Tsa2 controlled by the the Tsa1 promoter. Efficient protection from oxidative stress requires not only the right enzymatic activity (which Tsa2 exerts) but also a sufficient level of expression [20]. Two peroxiredoxin isoforms are even found in the nucleus (Dot5) and in the mitochondria (Prx1), respectively [23,24], showing that the detoxification of ROS by this system is important in these subcellular compartments [25]. Tsa1 activity has been shown to be necessary for suppressing genomic instability in the yeast *S. cerevisiae* [20]. This would logically point to the fact that oxidative stress defense in the cytoplasm somehow exerts influence on the redox reactions taking place in the nucleus. However, the mechanism which is at work here has not been investigated.

Six isoforms have been characterized in human cells [26], of which one (PrxI) is the ortholog of Tsa1and can functionally replace it as shown by genetic experiments [26].The human isoforms are named PrxI to PrxVI. PrxI and II are 2Cys peroxiredoxins located in the cytoplasm, PrxVI is a 1Cys isoform also located in the cytoplasm. PrxIII is a 2Cys enzyme located exclusively in mitochondria, and PrxV is an atypical 2Cys peroxiredoxin located in the cytoplasm, mitochondria and peroxisomes. Finally, PrxIV is a 2Cys peroxiredoxin located in the ER which functionally interacts with PDI (protein disulfide isomerase) in the redox reaction forming the disulfide bonds of proteins destined for the secretory pathway. This latter function of the PrxIV protein seems to be specific for the mammalian system and to be still undiscovered or absent from fungal cells.

**Figure 2:**
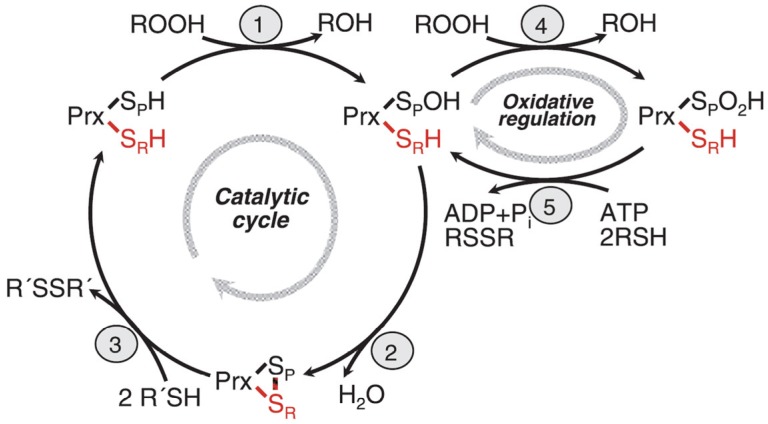
Reaction cycle of eukaryotic 2-Cys peroxidredoxins. Step 1: The peroxidatic cysteine is oxidized to the sulfenic acid and concomitantly substrate (ROOH) is reduced to ROH. Step 2: The sulfenic acid form of the peroxidatic cysteine reacts with the resolving cysteine in the other subunit of the dimer, forming a disulfide bridge and releasing water. Step3: The disulfide form reacts with its redox partner, thioredoxin yielding the fully reduced form which can start a new reaction cycle. Step 4: The sulfenic acid form of the protein is further oxidized by substrate to the sulfinic acid form which is catalytically inactive and forms a chaperonine. Step 5: The sulfinic acid form is reduced back to the sulfenic acid form. Its redox partner is sulfiredoxin which is this reaction forms a diulfide bridge. The reaction is energy dependent and consumes ATP which is hydrolyzed to ADP and P_i_. See text for further discussion of the reaction cycle, in particular the structural transition to form a chaperonine, and the local unfolding of the structure (after Hall *et al.* 2009 [19]; with modifications).

The catalytic cycle of eukaryotic peroxiredoxins involves a number of peculiarities explaining its regulation, recognition of multiple substrates, and role in H_2_O_2_ signaling. The protein undergoes physiologically important local unfolding during its reaction cycle [19,24,27]. We are discussing here only the 2Cys peroxiredoxins. The enzymes are active as homodimers associated in a head to tail arrangement where each subunit contains two unequal important cysteine residues, named the peroxidatic (catalytic) cysteine C_p_, and the resolving cysteine, C_r_. In the ground state, the protein is fully reduced (both cysteines in SH form) and fully folded (FF), the two cysteines which will from an intersubunit disulfide bond are still separated by about 14 Å, and the C_p_ residue is well shielded by a C-terminal helix and the GGLG loop. These two structural motifs occur only in eukaryotic peroxiredoxins which have developed an additional signal transmission function, but not in the prokaryotic peroxiredoxins. In the first step of the reaction cycle (Figure 2) both Cp residues of the homodimer are oxidized to the sulfenic acid level and the substrate is reduced (typically from ROOH to ROH), in the second step, local unfolding takes place with respect to the C-terminal helix and the GGLG loop. This unfolding enables the C_p_ of one subunit to approach C_r_ of the other one and formation of the disulfide bond with release of water. The –S-S- form is still locally unfolded (LU) and can now react with the redox partner, thioredoxin (also with other redox patner proteins which are not well known at present), to reform the ground state (fully reduced and FF). We assume that the local unfolding is essential for the catalytic activity, both by enabling formation of the intermediate disulfide form and by enabling the protein to interat with so many different substrates, but also with different interaction partner proteins.

The intermediate sulfenic acid state can be further oxidized by a second molecule of H_2_O_2_ (or other oxidized substrates). This is relatively easy to achieve leading to the hyperoxidized state containing the sulfinic acid form of C_p_.This form of the enzyme which is fully folded is catalytically inactive, and attains a new structure and activity. It is a decamer (a ring of five dimers) and, moreover, several of the rings form a hollow cylinder which is an efficient chaperonine re-folding misfolded proteins after inserting them into the hollow chamber. The sulfinic acid form can be reduced back to the sulfenic acid by the enzyme, sulfiredoxin, a reaction which needs the help of NADPH and ATP [28]. The physiological significance of this process in signal transmission will be discussed in the next paragraphs. Even a further oxidation of the sulfinic acid form to the sulfonic acid form has been observed. However, this process is thought to be irreversible *in vivo*.

## 3. Protein Disulfide Isomerase (PDI) in Oxidative Stress and Signaling

Protein disulfide isomerase (PDI) and the isoforms of this enzyme, like peroxidredoxins, are highly abundant redox proteins of eukaryotic (and prokaryotic) cells which are located in the endoplasmic reticulum (ER) of eukaryotic cells and are deeply involved in the creation and regulation of oxidative stress in mammalian as well as in fungal cells. Excellent reviews exist describing the structure and function of PDI [29,30,31]. These authors consider the ER as the main source of oxidative stress in the cell which can be produced as a consequence of the unfolded protein response (UPR). The direct source of ROS (in particular H_2_O_2_) in this mechanism is *ERO1* (an essential gene in yeast which has been studied intensively in relation to UPR) and supplies oxidation equivalents to PDI.

The primary function of the enzymatic pathway involving PDI (in *S. cerevisiae* the isoforms Pdi1, Eps1, Eug1, Mpd1 and Mpd2) and Ero1is threefold: (i) oxidation of cysteine SH groups during attainment of the correct folding of secreted proteins in the ER; (ii) reduction of disulfide bridges which are incorrectly formed; and, (iii), isomerization of disulfide bridges which are often formed in the incorrect place in multi-cysteine secreted proteins. The ultimate sources of oxidation and reduction are molecular oxygen which is transformed to H_2_O_2_ by Ero1, and NADPH, respectively. Not suprisingly, PDI protein domains are members of the thioredoxin fold superfamily.They can interact in redox reactions with a number of additional partner proteins [29]. PDI seems to play a central role in induction of apoptosis [30,32,33] which includes a signaling pathway consisting of pro-apototic and anti-apoptotic modules. It is unclear at present how the adaptive (*i.e.*, cell survival) and apoptotic (*i.e.*, cell death) branches of this pathway are balanced. Very unexpected steps are included, like externalization of PDI from the ER to extramitochondrial membranes [29]. At present, a conclusive picture of the role of PDI in oxidative stress in fungi is not yet emerging. However, there can be no doubt that in the near future more interesting facts about the function of PDI in oxidative stress and signaling will be discovered, using the well known fungal model systems *S. cerevisiae* and *S. pombe*.

## 4. Signaling through Hydrogen Peroxide and the Function of Peroxiredoxins as Modulators of Signaling

We would like to start this part of the chapter by giving examples of signaling through ROS or H_2_O_2_ in those cases where information about the signaling mechanism and the signaling partners is available. As has been stressed before, mammalian cells are in this respect much better known than fungal cells. However the mammalian examples can tell us what we can possibly expect. Another good example is supplied by *S. pombe*, a fungal system that is not closely related to *S. cerevisiae*. Both examples provide possible roadmarks for which to look in *S. cerevisiae* and other fungi.

In those rare cases where detailed information about the fungal systems is available, we can point out the differences to mammalian cell systems. In principle, redox signaling can be divided into three different possible and partly documented mechanisms [19] discussed in the examples given below.

*Example 1*: In this example from mammalian cells, an extracellular signal which is not itself a redox signal, is transformed into a redox signal in the cell addressing phosphotyrosine phosphatase 1B (PTP1B) [34,35]. An excellent review including PTP1B was published recently [13]. Generally, PTPs in their active site carry a low pK_a_ cysteine SH group which is prone to oxidation [36]. In the case described here, the non-redox signal (epidermal growth factor, EGF) is amplified and transmuted (discussed below) into a redox signal (H_2_O_2_), which transiently inactivates PTP1B by sulfenylation at the catalytic cysteinyl SH group of the enzyme [37]. This in turn leads to an increase in the tyrosine phosphorylation state of epidermal growth factor receptor (EGFR) which is a tyrosine kinase capable of autophosphorylation. This amounts to a feed-forward amplification of the signal. In this case, compartimentation of the H_2_O_2_ signal is reached because on binding of EGF to the EGFR, the latter is through endocytotic vesicular transport moved to the ER where it stimulates the ER-located Nox4 (in the relevant epithelial cell cultures) to produce H_2_O_2_. The hydrogen peroxide originating from Nox4 in turn sulfenylates the receptor tyrosine kinase as well as the PTP1B. The former is activated by sulfenylation while the latter is inactivated. Both effects lead to a strong feed-forward reaction. Production of Nox4 is transcriptionally controlled in this system by the EGFR signaling pathway. Although this system is in our view one of the best described in mammalian cell H_2_O_2_ signaling, many open questions obviously remain. Above all, it is unclear how the signal created in the ER is further transmitted reaching ultimately the trancription machinery in the nucleus. It is known that EGFR activates c-myc and CREB. An equally important open question is the eventual down-regulation of this powerful signaling. This awaits further research in the future. As the key mechanistic steps occur in the ER, it is quite probable that the ER-located peroxiredoxin, PrxIV, and the ER-located PDI play a significant yet still unknown role in this process. Of note, the only *S. cerevisiae* NADPH oxidase identified biochemically so far, resides in the ER, so that the suggested signaling function of YNO1 in reorganization of the actin cytoskeleton during the cell cycle [38] could in part follow the mechanistic model described above.

Other examples from mammalian cells can be found in the published literature [36,39,40,41].

*Example 2*: In this example, which has been mostly studied in yeast, a redox signal from outside is amplified in the cell to stimulate a defense response to oxidative stress by formation of a disulfide covalent linkage of peroxiredoxin to a partner protein resulting in a downstream transcriptional response [19]. Typically, as a result, a trancription factor forms an internal disulfide bridge which results in blocking nuclear export, transfer to the nucleus and activation of the downstream oxidative stress response genes. The example was discovered in *S. pombe* [42]. The transcription factor governing the low level of oxidative stress defense reaction in this yeast is Pap1, the structural and functional homolog of the well-known *S. cerevisiae* trancription factor Yap1 which governs oxidative stress response. In *S. pombe*, Pap1 is under control by the H_2_O_2_ sensor Tpx1, one of the *S. pombe* peroxidredoxins. A similar disulfide bond formation in *S. cerevisae* is indirect, with the primary H_2_O_2_ sensor being the glutathione peroxidase, Gpx3, which then in turn via a disulfide cascade creates a disulfide bond on Yap1. A second pathway depends in a similar way on oxidation of Sty1, a MAPkinase (MAPK), activating the downstream transcription factor Atf1, which is likewise involved in oxidative stress response. This pathway is important for survival of high levels of H_2_O_2_, while the Pap1 pathway is involved in adaptation to low levels of H_2_O_2_ [42]. Of note, these important *S. pombe* redox signaling pathways have up to now only been studied using external H_2_O_2_. The important question is, of course, what is the still unknown internal source of H_2_O_2_ for signaling. Does *S. pombe* display redox signaling also in cases where the primary signal is not external oxidative stress? Other examples from *S. pombe* exist but are not described in detail here due to space restrictions [43].

*Example 3*: In this example the authors [19,27] propose their floodgate model (more appropriately called the adjustable buffer model) for the role of the yeast peroxiredoxin, Tsa1, in the response to an H_2_O_2_ signal. Tsa1 deficiency [44] as well as increased unregulated activity [45] result in accelerated mother cell-specific and chronological aging in *S. cerevisiae*. The floodgate model is presently a very attractive one. The experimental findings are consistent with the model however still without detailed proof of the molecular mechanism in fungi.

The eukaryotic members of the peroxiredoxin protein family (but apparently not the prokaryotic ones) in addition to their defense function play an important role in hydrogen peroxide signaling, and suprisingly also as chaperones (when hyperoxidized), and are regulated mainly by the redox state of their active site cysteines, but also through phosphorylation [46] and other post-synthetic modifications like glutathionylation, and through a large number of partner proteins [47]. A general overview of redox-based modulation of signal transduction by peroxidredoxins is given by Janssen-Heiniger *et al.* [48] and Park *et al.* [47].

We want to discuss and make it plausible why H_2_O_2_, a molecule exerting considerable oxidative damage in cells, has nevertheless been chosen by “Mother Nature”, as a signaling substance. Signaling through H_2_O_2_ works well in eukaryotic cells (little is known about bacteria), because H_2_O_2_ is a stable non-radical substance occuring naturally through normal metabolic reactions that displays a sufficient half-life to be able to migrate (diffuse) for a few microns within the cell. H_2_O_2_ is electrically neutral and could diffuse through lipid bilayer membranes, however, in real life it is passing membranes bymeans of the aquaporin channels [49]. H_2_O_2_ is synthesized by “regular” metabolic reactions, by “leaky” electron transfer, but also by special reactions designed for the sole purpose of creating ROS. Several reactions come to mind and will be enumerated here, of which one (NADPH oxidases) will be discussed in more detail. It is reactive chemically towards its target proteins with the typical reaction being oxidation of a target SH group which is a reversible reaction. It acts locally (see below) without destroying cellular components outside the target area. It can be readily destroyed if no longer useful by a number of detoxification pathways (see Figure 1) which is essential for every signaling substance. Eukaryotic peroxidredoxins are involved in both the destruction of H_2_O_2_ and in its transient stabilization through hyperoxidation of the peroxidatic sulfhydryl group (Figure 2).

Prokaryotic peroxiredoxins cannot be inactivated by high hydrogen peroxide [24]. This resistance to high hydrogen peroxide is accompanied by absence of the flexibility of the C-terminus and around the resolving Cys. Therefore, the prokaryotic enzymes are not physiologically inactivated by hydrogen peroxide like the eukaryotic ones. Probably this means that the prokaryotic peroxiredoxins are not involved in signaling, only in oxidative stress defense.

Peroxiredoxin is a secondary modulator of eukaryotic hydrogen peroxide signaling. The producer of H_2_O_2_ is (among other enzymes) typically an NADPH oxidase, in conjunction with a superoxide dismutase (SOD). The two enzymes may be tightly linked in the cell [16,40]. In a localized burst of H_2_O_2_, peroxiredoxin is locally hyperoxidized, as already mentioned above, leading to inactivation as a peroxidase and enhancement of the H_2_O_2_ signal and to a new function as a chaperonine.

The target of the H_2_O_2_ signal is most often a phosphotyrosine phosphatase (PTP) [36], as has been described above in Example 1, but is not yet investigated in detail in fungal organisms. PTPs in turn influence protein phosphorylation through protein kinases, which are often key modules of cellular signaling. In order to do this, a certain minimum local concentration of H_2_O_2_ must be attained, which is around 10 mM while the typical, maximum bulk concentration of H_2_O_2_ measured in higher cells is about 0.1 µM (in resting cells) and 0.7 µM (after stimulation through a signal), four to five orders of magnitude lower [50]. Bulk intracellular H_2_O_2_ concentration above 0.7 µM lead to apoptosis [50]. It is, therefore, clear that the burst of signaling H_2_O_2_ must be strictly confined in time and space in the cell, unless unwanted oxidative stress reactions are elicited in the cell. This is achieved by the peroxidredoxin system which is the principal hydrogen peroxide degrading system in the cytoplasm. It efficiently removes the signaling substance after the signal has been transmitted, and it does not do it as long as peroxiredoxin is transiently inactivated by oxdizing the catalytic SH group to the inactive sulfinic acid state as mentioned above. Sulfiredoxin (Srx) reduces the sulfinic acid back to the thiol in an ATP and thioredoxin or glutathione-dependent reaction, thereby completing the oxidative regulation cycle of peroxiredoxin [51,52,53,54,55]. This means [19] the reversible opening and closing of a gate or buffer for H_2_O_2_.

## 5. Metabolic Reactions Generating H_2_O_2_

Several sources of H_2_O_2_ in fungal cells (and in higher cells) have been found. There are several obvious possibilities for the production of H_2_O_2_ in the metabolism of fungal cells. They have been listed in the literature: glyoxal oxidase and aryl alcohol oxidase [56], and the combined action of one electon transfer to oxygen in the respiratory chain of mitochondria in conjunction with superoxide dismutase (MnSOD) [57,58]. A further source of H_2_O_2_ is the combined action of NADPH oxidases with SOD which is highly regulated in space and time [16,40]. For the occurrence of NADPH oxidases in fungi, the reader is referred to the discussion of “Fungal NADPH Oxidases” below. Another source of H_2_O_2_ is PDI (protein disulfide isomerase), which in fungal cells like in all eukaryotes occurs in the endoplasmic reticulum and may be quantitatively more important for ROS production than the mitochondria [29,31]. The role of NADPH oxidases in signaling has up to now not been researched intensively in fungal systems as much as it has been in mammalian cells.

## 6. Fungal NADPH Oxidases and Their Function in Cell Differentiation

The first report of a fungal NADPH oxidase goes back to Lara-Ortiz *et al.* [59]. In the beginning of research on fungal NADPH oxidases, the only well-known example of an NADPH oxidase (NOX enzyme) was the human enzyme, NOX2, which is located in the plasma membrane of macrophages/monocytes and plays an important role in non-specific defense against bacterial and fungal infections. Therefore, research concentrated on true orthologs of NOX2 which were expected to exist in fungal cells [60,61,62]. This means that at that time researchers were trying to find not only orthologs of the human defense enzyme but also orthologs of its regulatory subunits, which was in part misleading because the fungal NADPH oxidases reside in different branches of the evolutionary tree of IMR (integral membrane reductase) enzymes [63,64,65] and are also regulated in different ways—compare the part on sequence-based evolutionary trees of fungal NADPH oxidases. Human Nox2, also called gp91^ph^°^x^, is located in the plasma membrane and regulated at the enzyme level by regulatory subunits which are cytoplasmic in unstimulated cells but transferred to the plasma membrane after stimulation of the macrophage cells. More than six regulatory interaction partners of pg91phox are known in the macrophage (and also in other human cell types), of which only two share homology with corresponding regulatory subunits of NADPH oxidase enzymes in fungi: NoxR (corresponding to p67^ph^°^x^) and the small GTPase, rac [62]. The function of NoxR in fungi is underscored by the mutant phenotype, for instance in *A. nidulans*, which is similar to the phenotype of the NoxA deletion, resulting in deficiency of cleistothecia formation. However, members of the NoxR family of fungi occur also in species that do not obviously contain orthologs of the classical fungal NADPH oxidases, and therefore presumably have additional new functions unrelated to Noxs. In filamentous fungi, the two classical protein families, NoxA and NoxB (in some fungi called Nox1 and Nox2) both show interaction with NoxR. Additional regulatory subunits are assumed to exist (based on the recognizable protein interaction domain on NoxR). Interactors were genetically identified in *Epichloe festucae* and found to be BemA and Cdc24, homologs of which are known to be involved in polarity establishment in fungal cells, and consequently in hyphal growth [66]. The physiological functions of the two enzymes, NoxA and NoxB, were found by analyzing the phenotype of the corresponding deletion mutants, and in some cases by screening for extragenic suppressors and by studying the action of NADPH oxidase inhibitors (for instance, diphenylideneiodonium chloride). In all cases, the mutant phenotypes were either a defect in cell differentiation (cleistothecia formation), so that these strains were female-sterile (NoxA deficiency, [62]), a defect in spore germination (Noxb deficiency, [67,68]), or a defect in the symbiotic interaction with a host plant in the case of plant parasitic or mutualistic fungi (NoxA deficiency [69]). The filamentous fungi studied most carefully in this respect were *Aspergillus nidulans* [59], *Podospora anserinum* [67,68,70], *Neurospora crassa* [71], *Epichloe festucae* [66,69], and others. In none of these cases, the molecular details of signaling which would explain the mutant phenotypes, are known [62]. However these findings are clearly in line with the general idea that ROS in fungi serve both as sources of oxidative stress, defence purposes (in the interaction with plants), and signaling to induce cell differentiation.

The third classical NADPH oxidase of fungi, NoxC (also called Nox3 in some species), does not interact with NoxR, carries two or more EF-hand calcium ion binding domains (like human Nox5) pointing to calcium regulation, is sequence–wise not closely related to either NoxA or NoxB, and functionally only known in exceptional cases, mostly in plant pathogenic fungi [62].

**Figure 3:**
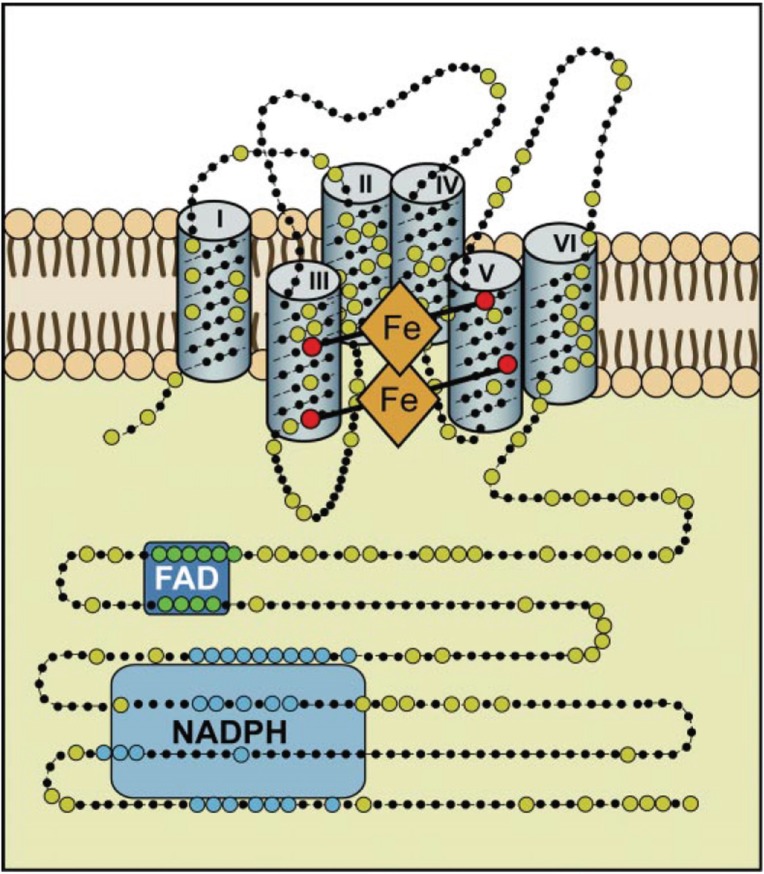
Hypothetical structure of a typical NADPH oxidase. This structural model is based on bioinformatics, cell fractionation and biochemical data concerning the human Nox enyzmes (NOX1, 2, 3, and 4); no crystallographic or NMR structural data are available yet. Nox enzymes comprise typically about 500 amino acids and are exclusively located in lipid bylayer membranes, like the plasma membrane or the ER membrane. Large dots are highly conserved amino acids. The reaction center transferring a single electron to oxygen is the upper b-type heme in this scheme. The enzyme consists of six transmembrane helices. The two b-type hemes are coordinated with histidine residues between helices III and V. The enzyme contains binding sequences for NADPH as well as for FAD in its cytoplasmic tail (after Bedard and Krause 2007 [60]).

NADPH oxidases are without exception located in biological membranes. They comprise 6 or 7 transmembrane helices, and produce superoxide in a vectorial way so that superoxide (in the example of the macrophage enzyme) is produced on the extracellular side of the plasma membrane and molecular oxygen and NADPH are consumed in the cytoplasm. The unique reaction catalyzed by NADPH oxidases is in need for three different cofactors: NADPH, FAD, and (two different) b-type cytochromes, as well as the substrate, dioxygen (Figure 3).The reaction equation can be summarized as: NADPH + 2O_2_ →NADP^+^ + 2O_2_^−^ + H^+^.

Superoxide itself is a well known oxygen radical which produces severe oxidative stress and oxidative damage to nearly all cellular components mostly through the formation of follow-up products, the most important of which is the highly reactive OH radical. In the defense reactions of macrophages, NADPH oxidase cooperates with myeloperoxidase and other peroxidases, forming a set of very highly active bactericidal compounds, like the hypochlorite anion, peroxonitrite, and others.

**Figure 4:**
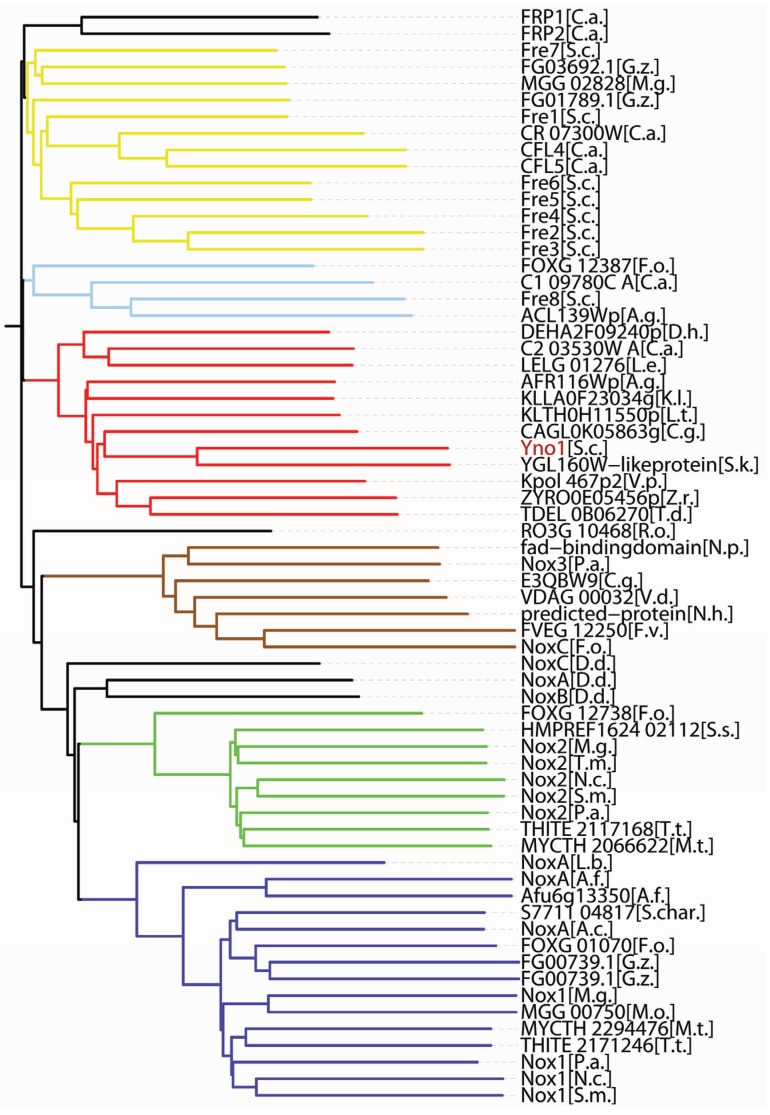
Phylogenetic relationships among the fungal members of the IMR (integral membrane reductase) protein superfamily (see also text for a discussion).

However, the picture of NADPH oxidases as a class of defense enzymes has been greatly changed and enlarged in recent decades and our present view of this class of enzymes now includes catalysis of specialized chemical reactions and also signal transduction. One example is synthesis of the biologically active form of the hormone, thyroxine, by DUOX2 and a thyroidal peroxidase in the thyroid gland in a radical reaction using iodide and H_2_O_2_ [60,61]. Signal transduction is another more general and more important new function of NADPH oxidases based on the production of ROS as signaling compounds which can signal cell proliferation but also cell differentiation [13]. The best available evidence for a signaling ROS exists for hydrogen peroxide in human cells as well as fungal cells. Signaling by NADPH oxidases in fungi [62,67] was studied in detail in connection with cell differentiation in *Aspergillus* [59], *Podospora* [70], and *Neurospora* [71]. These examples relate to the formation of fruiting bodies needed for sexual reproduction, spore germination or interaction with a plant host and without exception concern mutations in the classical fungal NADPH oxidases NoxA, NoxB, and NoxC or the regulatory fungal NOX subunit, NoxR, of filamentous fungi (see also the cladogram given in Figure 4). The mutant phenotypes are pronounced, leading, for instance, to female sterile mycelia. However, there is presently no information available on the molecular mechanisms which would explain how these fungal NADPH oxidases or the ROS produced by them are involved in the physiological cell differentiation in fruiting bodies.

The NADPH oxidase of *S. cerevisiae*, Yno1 [38] is not closely related to the classical fungal NADPH oxidases NoxA, B, and C, is located in the ER and was studied by *in vitro* biochemical activity determination. The deletion of the gene confers no defect in cell differentiation, but leads to hypersensitivity to antibiotics inhibiting the actin cytoskeleton. Subsequent work [16] showed that Yno1 is directly coupled to the superoxide dismutase, SOD1, so that the desired signaling substance H_2_O_2_ is tightly controlled in space (and time) leaving no possibility for the primary product, superoxide, to engage in other, deleterious or unwanted metabolic pathways. The topic of ROS sequestration is actively researched in many experimental systems, also in higher cells. Toledano and co-workers [40] argue that hydrogen peroxide as a signaling molecule requires protection of those proteins which are not immediately involved in the signaling process. The physiological endpoint found by Reddi and Culotta [16] is the regulation of mitochondrial respiration depending on carbon source and growth phase. This work and the work by Leadsham *et al.* [72] clearly shows that Yno1’s function becomes most obvious when yeast cells use up glucose and reach the point of diauxie. Under these conditions, certain respiratory defective mutants induce a burst of ROS which is under control of Ras2 activity leading to apoptosis, however, is completely abolished in the *Yno1* deletion mutant. This finding would indicate a function for Yno1 in controlled cell death of yeast.

The cladogram shown was calculated (Vector NTI^®^ Software Package, Life Technologies, Carlsbad, CA, USA) using 60 representative fungal IMR protein sequences available in the sequence databanks (http://www.ncbi.nlm.nih.gov/gene) by December 2014. The length from the (calculated) origin of the tree to each protein sequence is proportional to the number of amino acid exchanges. The resulting phylogenetic tree is clearly divided into six subfamilies which are separated from each other by deep valleys. In addition, a small number of outliers are shown, which are not members of the six subfamilies. They are discussed in the text. Starting from the bottom, the subfamilies are: **blue:** NoxA or Nox1 (please note that there is no agreed unified nomenclature and researchers working with different fungal species have invented different names for the enzymes), in some but by no means all proteins of the NoxA group, *in vitro* biochemical experiments have shown NADPH oxidase activity–the same is true for NoxB and NoxC; **green:** NoxB or Nox2; **brown:** NoxC or Nox3; **red:** the subfamily of Yno1 homologs, as discussed in the text, *in vitro* biochemical activity measurements clearly show NADPH oxidase activity for the founding member, *S. cerevisiae* Yno1 (shown in red), the other (non-cerevisiae) members of the group were not tested biochmically, but they could be NADPH oxidases due to their close sequence similartiy to Yno1; **light blue:** the Fre8 group, *S.c.* Fre8 (but not the other FRE genes) showed weak NADPH oxidase activity, the other three members were not tested; **yellow:** the ferric reductase subfamily, some, but by no means all of the members were tested for biochemical activity reducing ferric iron complexes during the iron uptake process in *S. cerevisiae*, the subfamily members from other fungi were mostly not tested.

Only the gene names used in the sequence databases are used in the figure.

Abbreviated species names (in alphabetical order) in the cladogram:

A. g. Ashbya gossypii
A. c. Acremonium chrysogenum
A. f. Aspergillus fumigatus
C. a. Candida albicans
C. g. Candida glabrata
D. d. Dictyostelium discoideum
D. h. Debaryomyces hansenii
F. o. Fusarium oxysporum
F. v. Fusarium verticillioides
G. z. Gibberella zeae
K. l. Kluyveromyces lactis
L. e. Lodderomyces elongisporus]
L. b. Laccaria bicolor
L. t. Lachancea thermotolerans
M. g. Magnaporthe grisea
M. o. Magnaporthe oryzae
M. t. Myceliophthora thermophila
N. c. Neurospora crassa
N. h. Nectria haematococca
N. p. Neofusicoccum parvum
P. a. Podospora anserina
R. o. Rhizopus oryzae
S. c. Saccharomyces cervisiae
S. char. Stachybotrys chartarum
S. k. Saccharomyces kudriavzevii
S. m. Sordaria macrospora
S. s. Sporothrix schenckii
T.d. Torulaspora delbrueckii
T. m. Togninia minima
T. t. Thielavia terrestris
V. d. Verticillium dahliae
V.p. Vanderwaltozyma polyspora
Z. r. Zygosaccharomyces rouxii

NADPH oxidases produce superoxide as a primary product, which is in many cases in living cells is the source of deleterious reactive oxygen species (ROS). Strict compartmentalization as well as regulation of enzyme activity and “channeling” of the radical through immediate interaction with SOD have led to the modern picture of NADPH oxidases involved in signaling. Some of the known examples of ROS signaling in fungi are presented in the present paper. It is attempted to cover the literature up to 2014. However, it also seems clear that the same NADPH oxidase systems whose primary role is signaling, can, under certain pathological conditions, be also a source of oxidative stress. This was first discovered and described in mammalian cells [73].

## 7. Sequence-Based Phylogenies of IMR Proteins

We now come to the question of sequence-based phylogenies of the NOX/IMR protein superfamily in fungi in relation to the biochemical function of these enzymes. IMR (integral membrane protein) is an acronym coined by Grissa *et al.* [63] which encompasses membrane proteins of similar sequences including NADPH oxidases and ferric reductases, and very probably enzymes with further still undiscoverd biochemical activities. There appear to be two well-separated branches in the sequence-based phylogeny of this protein superfamily as calculated by Grissa *et al.* [63], with all the true NOXes known at the time falling into one branch of this phylogeny and the ferric reductases (integral membrane reductases) into a separate one. However, we showed by *in vitro* biochemical methods that *YNO1*, located in the published phylogeny in subfamily XVII of IMR proteins together with *FRE8* (showing only a small superoxide producing capacity), is a *bona fide* NADPH oxidase, while the other *FRE* genes of yeast (*FRE1* through *FRE7*) are not [38]. We cannot, therefore escape the conclusion that at least one subfamily of IMRs, which is attributed to the ferric reductase branch by Grissa *et al.* [63], codes not for ferric reducase gene products, but for a biochemically proven NADPH oxidase.

This is further documented in Figure 4. To illustrate the YNO1 subfamily as defined in Figure 4 even more, we have systematically compared the YNO1 sequence over its whole length with close relatives from *C. albicans* and *C. glabrata*, as well as with its *S. cerevisae* paralogs located in the *FRE* subfamily. Yno1 and the best match from *C. glabrata* (CAGL0K05863g) share 40.9% identity and 69.9% similarity. By the same criteria, Yno1and its paralog Fre1 share only 18.2% identity and 47.8% similarity. By the way, Yno1 shares only a weak identity and similarity with the typical members of the fungal NoxA, B, and C proteins. Apparently, the same biochemical activity, NADPH oxidase, can be reached in several quite different subfamilies of the large IMR protein superfamily.

It would be interesting to compare the three-dimensional (3D) structures of NOX and ferric reductase enzymes with the one of Yno1 to see if, perhaps, the Yno1 structure is more closely related to the NOXes than to the ferric reductases. Protein structures generally show a stronger conservation and correlation with function, than sequence alone. Unfortunately, X-ray crystallography has so far not yielded any structures of the membrane–bound IMR proteins. The argument by Lalucque and Silar [74] linking fungal NOX enzymes with multicellularity would correlate well with *S. cerevisiae* and *S. pombe* having no NOX enzymes (based on sequence criteria alone), while *P. anserina*, *A. nidulans*, and many other filamentous fungi do contain coding sequences for NOXA, B, and/or C enzymes. However, the monocellular *S. cerevisiae* yeast does contain a *bona fide* NOX enzyme, so the generalization that Noxes are enzymes of multicellularity [74] is certainly no longer true. One could, however, argue that *S. cerevisiae* is a close relative of a multicellular filamentous fungal plant parasite (*A. gossypii*) and was probably derived from an ancestral plant parasite in the not too distant evolutionary history [75] which could possibly explain the presence of a *bona fide* NADPH oxidase in this yeast.

Very highly similar sequences to the one of Yno1 exist in *C. albicans* as well as in *C. glabrata* (see Figure 4). *C. albicans* is a dimorphic human pathogen and hyphal growth seems to be necessary for pathogenicity. The phenotype of the homozygous deletion mutant of the *C. albicans* Yno1 ortholog (C2 03530W A) is currently being investigated and should shed additional light at the physiological functions of this group of fungal NADPH oxidase enzymes.

## 8. Oxygen Radicals in the Interaction of Plants with Their Fungal Symbionts and Parasites

A newly emerging field is the long-distance signal tranduction in plants interacting with their fungal symbionts and parasites and likewise the response elicited in the fungal cell during the successful invasion leading to mutualistic or parasitic interaction with the plant. This phenomenon will be viewed here only from the perspectice of the fungal cell.

The mechanism of signal transduction in plants is only now beginning to be understood. ROS and calcium ions play a major role in this process [76]. In this field, a major aim is to gain knowledge about plant NADPH oxidases which are the most important sources of ROS for signal transduction [77]. For instance, in *A. thaliana* 10 different Rboh (respiratory burst oxidase homologue) genes exist which are called Atrboh and have specialized functions for the life cycle and defense of this plant. A more specialized update was given by Torres *et al.* [78] concerning ROS signaling of the plant in response to pathogens.

A finding that is fascinating and not understood at all at the present time is that although plant defense relies heavily on NADPH oxidases, the so-called Rboh enzymes, the fungi also need NADPH oxidases for the successful invasion of and interaction with the plant host. In most cases, the NoxA, B or C enzymes are concerned.

A good example is the infection of rice leaves by *Magnaporthe grisea*. Both the NoxA and the NoxB genes of the fungus are needed for the succesful penetration, in particular for the formation of the appressorium. The respective deletion mutants of the two Nox genes have lost virulence and the defect is in the infection process [79].

Another example is known in somewhat more molecular detail. NoxA activation through rac is required to establish a mutualistic symbiotic association between *Epichloe festucae* and its host, the perennial ryegrass *Lolium perenne* [66,69,80,81]. The fungal hyphae grow in the extracellular space of the plant. In the absence of NoxA, *in planta*, the hyphae overgrow and branch and eventually kill the plant, which shows stunted growth. If the fungus is grown on agar plates in the absence of plants or plant materials, no strong phenotype of the NoxA deletion mutation is shown. Obviously, the mutualistic relationship between plant and fungus requires a delicate balance of redox signaling which is not nearly understood at present.

## 9. ROS Production and the Degradation of Lignocellulose by Fungi

In a short review article [82], the current status of biofuel production from lignocellulose is described. The US this year opened the first factories which produce bioethanol from cellulose (corn stover) in large quantities (more than 300 million liters per year at their present capacity). However, the first step is still the energy demanding conversion of cellulose and hemicellulose to sugars by purely chemical and physical means (heat and NaOH). The true breakthrough will be the biological conversion by fungi or fungal enzymes (white rot fungus, and others) which can degrade lignocellulose at room temperature and physiological pH. This process consists of radical reactions and is absolutely dependent on the enzymes, lignin peroxidase and laccase, and on ROS produced in the fungi by glyoxal oxidase, aryl alcohol oxidase, and NADPH oxidases. These processes are up to now poorly known to mycologists and biotechnologists but are under intensive research presently. If we know more about the natural processes, another big challenge will be the design of a commercial process based on the degradation of lignocellulose.

Surprisingly, both the degradation of lignin and of cellulose which are covalently linked in wood (through hemicellulose), require oxidative steps. Both extracellular and intracellular enzymatic reactions are required.

Cellobiose dehydrogenase (reviewed in Baldrian and Valaskova [83]) is described as an example of oxidative degradation. The disaccharide is oxidized at the reducing end C1 atom to the corresponding gluconolactone, and is subsequently converted to the open chain gluconic acid. The enzyme uses FADH_2_ and b-type cytochrome as redox co-enzymes and interacts with cytochrome c and/or quinones in one electron transfer reactions. The ultimate source of oxidation equivalents for this reaction is presumably H_2_O_2_.

The degradation and separation of lignin from cellulose in biotechnological processes presently is still done chemically. It serves the purpose of making cellulose more accessible to the enzymes degrading it. The degradation of lignin in nature is cost-efficiently performed by basidiomycete white rot fungi, and *Phanerochaete chrysosporium* is well researched with respect to this process [84].

Laccases are an important class of enzymes that are needed in plants for lignin synthesis and in fungi for lignin degradation [84]. Laccases are extracellular agents catalyzing crucial steps in lignin degradation by fungi. These well studied 4Cu enzymes (“blue enzymes”) reduce dioxygen to water in one-electron steps creating phenolic radicals (for instance semihydroquinones) leading to cleavage of C-C bonds in the phenylpropanoid subunits of lignin (so far shown only using soluble lignin model compounds), thereby cleaving the lignin macromolecule to smaller molecules. Laccases accomplish lignin degradation in conjunction with the peroxidases discussed in the next paragraph, but they are not *per se* essential for ligin degradation, as for instance the white rot fungus *Phanerochaete chrysosporium* does not contain a recognizable laccase-encoding gene in its genome sequence. The action of laccases creates considerable oxidative stress in the vicinity of fungal cells growing on wood. H_2_O_2_ is not involved in the known mechanism of the laccase reaction.

## 10. Lignin Peroxidases (LiP) and Manganese Peroxidases (MnP)

Apparently these peroxidases play an essential role in the extracellular degradation of lignin by fungi, as suggested by genetic and genomic data [84]. Peroxidases are heme proteins and employ H_2_O_2_ as a substrate, form an oxo-ferryl compound (recognized spectroscopically) in a first step, thereby reducing H_2_O_2_ to H_2_O; and a compound II in a second step, in which one electron is transferred to an aromatic non-phenolic structure of lignin. Thus, degradation by a radical mechanism is started. Most frequently, bonds in the side chains of phenylpropanoid units are broken leading to a variety of small molecule products (examples are a number of benzoic acid derivatives).

Manganese peroxidases (MnPs) also use H_2_O_2_ as a substrate, but oxidize Mn^2+^ to Mn^3+^, a highly oxidizing diffusible reagent which is believed to help degradation of sterically hindered moieties of lignin. A third type of extracelllular peroxidases (versatile peroxidases or “novel peroxidases”, NoP) have also been found but are less well researched. The genome of *P. chrysosporium* contains 10LiP, 5 MnP and one NoP-encoding genes [85].

## 11. The Use of Fenton Chemistry by Wood-Degrading Fungi

It is remarkable that processes like the Fenton and Haber-Weiss reactions are employed in the life cycle of fungi growing on wood, which produce highly toxic and aggressive molecular products like the hydroxyl radical and are avoided as much as possible in the cellular metabolism of prokaryotes as well as eukaryotes. Consider the careful avoidance of free ferrous ions in living cells, which are complexed in cellullar stores where they cannot take part in the unwanted production of oxygen radicals. The same applies to the Cu^+^ ions which in a similar way can support the Fenton reaction [86]. In the case of the degradation of wood by fungi, the “strong” chemistry of the Fenton reaction seems to be a way to break up lignin and recycle biologically the enormous amount of dead biomass of woody plants. The process is of course extracellular and highly controlled (see below). A detailed knowledge of this process and its regulation would be highly desirable for the development of the “second generation” of biofuels which requires the degradation and fermentation of the non-edible waste parts of plants, like for instance corn stover. In a further step to be taken in the future, even the woody parts of trees which remain in millions of tons in the timber industry processing of trees, could be used.

The strategy to use Fenton chemistry for lignin degradation is mainly used by the basidiomycete brown rot fungi, which express less extracellular peroxidases than the white rot fungi and digest mainly the cellulose part of wood. The fungi which were mainly studied in this respect are *Gloeophyllum trabeum* [87] and *Postia placenta* [88]. In order to produce sufficient activity and amounts of hydroxyl radicals, these fungi have invented two additional strategies: First, the secretion of either one or both of two quinone compounds which are efficient redox cyclers capable of one-electron transfer reactions and necessary for the extracellular generation of superoxide for the Fenton process: these are 2,5-dimethoxy-1,4-benzoquinone (2,5 DMBQ) and 4,5-dimethoxy-1,2-benzoquinone (4,5 DMBQ). Both compounds are produced as downstream metabolites of lignin degradation [87,89]. Second, these fungi secrete oxalic acid (also produced from lignin degradation metabolites) which chelates iron in the extracellular space [90]. The chelated Fe^2+^ ions are susceptible to oxidation and support the key Fenton reaction with H_2_O_2_. The process is depicted in Figure 4 of Bugg *et al.* [84]. A very similar but quantitatively less important Fenton process is used also by white rot fungi [91].

All of the processes described so far, with the single exception of the laccase reaction, require H_2_O_2_. The known sources of H_2_O_2_ in fungal cells have been listed above in the part on Metabolic Reactions Generating H_2_O_2_.

## 12. Further Degradation Products of Lignocellulose

Enzymatic breakdown of cellulose and hemicellulose is straightforward leading to glucose and other monomeric sugars and sugar derived metabolites (like gluconic acid, see above). These metabolites are used as carbon sources by the wood-rotting fungi, if no other carbon sources are available, and are extremely valuable for use in a new generation of bio-ethanol production facilities [82]. Lignin degradation is thought to be (at least in part) necessary to enable the attack of lignocellulose by extracelllular cellulases.

The processes which can solubilize and liberate small molecules representing partial structures of lignin are well researched and include a large variety of structures which are all rationally derived from the phenylpropane building blocks of this irregular and cross-linked polymer [84]. Most important are benzoic acid, benzaldehyde, cinnamic acid, substituted biphenyls, substituted diphenyl ethers, acetophenone, and many others, justifying the attempts to create novel bio-refinery processes and extracting industrially valuable chemicals. However, these downstream processes are not the subject of the present review paper.

## 13. Conclusions

We have, in this study, attempted to give an overview of three special aspects of oxidative stress in fungi: ROS as signaling molecules and NADPH oxidases as a major source of ROS; the role of ROS in the interaction of fungi with their plant hosts; and the extracellular degradation of lignocellulose by fungi utilizing ROS for this purpose. In the kingdom fungi, like in animals and plants, oxidative stress is both a substantial challenge for cellular survival that must be overcome by appropriate defense systems, but also something that can be used for pro-survival purposes in the specific situations of fungal cells. Several highly developed uses of oxygen radicals, ROS and the generation of oxidative stress come to mind and were reviewed above:

(i) The use of ROS (in this case H_2_O_2_) for intracellular signaling in the decision between growth and proliferation on the one hand, and growth arrest and cell differentiation on the other. This kind of signaling has to be compared with H_2_O_2_ signaling in mammalian cells, where much more information is available and the parallels but also the differences between the two signaling systems must be clarified.
(ii) The interaction of fungi with their plant hosts, both in parasitic and symbiotic relationships.
(iii) The degradation of lignocellulose, which is an environmental process of overriding importance for homeostasis in the biosphere. Lignocellulose can only be degraded in nature if oxygen radicals attack this highly resistant polymeric structure. The process must therefore take place in the extracellular space and the cells which use this process for supplying carbon sources for growth must, on the other hand, protect themselves against its detrimental consequences.

While it was traditionally thought that one electron transfer processes occurring erroneously in the mitochondrial respiratory chain are the main or only intracellular source of ROS (apart from such specialized systems as mammalian macrophages), it is now clear that ROS (superoxide radical anion and H_2_O_2_) are formed “on purpose” in every cell, by NADPH oxidases. We have therefore included a discussion of the present state of research on fungal NADPH oxidases.

Defense systems against oxidative stress are numerous and highly conserved in all eukaryotic and even prokaryotic cells. Many of the molecules, used normally for defense, can also play a role in signaling. As foremost examples we have discussed here the function of peroxiredoxins and of protein disulfide isomerases in fungal cells.

Taken together, the study of fungal oxidative stress, its use in the life cycle of fungi and its importance for cycles of matter in the biosphere, are a presently intensively researched topic and will be so even more in the future.
